# Isolation of Mitochondria From Fresh Mice Lung Tissue

**DOI:** 10.3389/fphys.2021.748261

**Published:** 2021-11-30

**Authors:** Dayene de Assis Fernandes Caldeira, Dahienne Ferreira de Oliveira, João Paulo Cavalcanti-de-Albuquerque, Jose Hamilton Matheus Nascimento, Walter Araujo Zin, Leonardo Maciel

**Affiliations:** ^1^Carlos Chagas Filho Institute of Biophysics, Federal University of Rio de Janeiro, Rio de Janeiro, Brazil; ^2^Institute of Medical Biochemistry, Federal University of Rio de Janeiro, Rio de Janeiro, Brazil; ^3^Professor Geraldo Cidade Campus, Federal University of Rio de Janeiro, Duque de Caxias, Brazil

**Keywords:** lung mitochondria isolation, O_2_-consumption, ROS, ATP, mitochondrial assessment

## Abstract

Direct analysis of isolated mitochondria enables a better understanding of lung dysfunction. Despite well-defined mitochondrial isolation protocols applicable to other tissues, such as the brain, kidney, heart, and liver, a robust and reproductive protocol has not yet been advanced for the lung. We describe a protocol for the isolation of mitochondria from lung tissue aiming for functional analyses of mitochondrial O_2_ consumption, transmembrane potential, reactive oxygen species (ROS) formation, ATP production, and swelling. We compared our protocol to that used for heart mitochondrial function that is well-established in the literature, and achieved similar results.

## Introduction

The assessment of mitochondrial function in organs and tissues is essential for a better understanding of their biochemistry, physiology, and pathophysiology (Weissig, 2005; Picard et al., 2011; Meyer et al., 2017; Murphy and Hartley, 2018). The evaluation of mitochondrial function is usually accomplished in isolated mitochondria (Picard et al., 2011; Gedik et al., 2017; Maciel et al., 2020, 2021; Caldeira et al., 2021) permeabilized fibers (Perry et al., 2013) or cells (Perry et al., 2013). These techniques are very well-defined in several types of tissue, e.g., heart (Gedik et al., 2017), kidney (Schulz et al., 2015), liver (Goudarzi et al., 2018), adipose tissue (Matta et al., 2021), and brain (Marques Neto et al., 2020), presenting peculiarities and different indications depending on the tissue and the purpose of the investigation. However, assessment of lung mitochondrial function presents difficulties associated with obtaining isolated, intact, coupled, and functional mitochondria. The methodological difficulty of obtaining viable lung mitochondria derives mainly from an elevated fatty acid content, low amount of mitochondria in the cell, fibrous and air-filled tissue, and the required amount of tissue (Spear and Lumeng, 1978; Kuznetsov et al., 2008; Lanza and Sreekumaran Nair, 2009). Recently, we have shown that the isolation of pulmonary mitochondria with preserved structure and function is possible by means of adaptations of existing techniques and standardization of a specific method of isolation by differentiated centrifugation (Caldeira et al., 2021). However, the differences related to mitochondrial isolation procedures for obtaining these well-preserved pulmonary mitochondria have not been addressed in detail, and the characteristics of reagents and equipment have not been fully described (Caldeira et al., 2021). Therefore, the main objective of this article is to provide a practical step-by-step user protocol upgraded to isolate pulmonary mitochondria. Our isolation protocol is founded on the differentiated centrifugation method of mice lung homogenate. However, unlike the classical procedures currently in use, we will use some innovative steps because of the intrinsic characteristics of the tissue to obtain better and more functional isolated mitochondria. In addition, we describe in detail the mitochondrial function pertaining to several respiratory complexes.

## Materials and Equipment

### Materials and Reagents

1. Teflon beaker (BRAND^®^ beaker, PTFE, low form, catalog number: Z322660; Merck, Darmstadt, Germany).
2. 50 ml Falcon tubes (FALCON^®^ Brand, 50 ml polypropylene conical tube 30 mm × 115 mm style, catalog number: 352070; Coring Science Mexico, Col del, Mexico).
3. 14 ml round-bottom tubes (Thermo Scientific™, Nunc™ 14 ml round-bottom tube, catalog number: 150268; Thermo Fisher Scientific™, Waltham, MA, United States).
4. 1.5 and 2 ml microfuge tubes (Eppendorf Safe-Lock Tubes, 1.5 and 2 ml Eppendorf Quality™, catalog numbers: 0030120086 and 0030120094, respectively; Eppendorf, Hamburgo, Germany).
5. Syringe filter (Corning^®^ syringe filters, nylon membrane, diameter 25 mm, pore size 0.2 μm, catalog number: CLS431224; Merck, Darmstadt, Germany).
6. Adjustable volumetric pipettes: 10 and 100 μl; and 5 ml (PIPETMAN L P10L, 1–10 μl; PIPETMAN L P100L, 10–100 μl; PIPETMAN L P1000L, 100–1,000 μl; PIPETMAN L P5000L, 500–5,000 μl, metal ejector, catalog numbers: FA10002M, FA10004M, FA10006M, and FA10007, respectively; Gilson, Middleton, WI, United States).
7. Dispenser pipette (BRAND^®^ pipette withdraw volume 3.5 ml, catalog number: 747755; Merck, Darmstadt, Germany).
8. Hamilton syringe: 10 and 25 μl (Hamilton^®^ TLC syringes. catalog number: Z264385 and Z264393, respectively; Merck, Darmstadt, Germany).
9. Potter-Elvehjem PTFE pestle and glass tube (catalog number: P7859; Sigma-Aldrich, San Luis, MO, United States).
10. Silica glass cuvettes (Starna Scientific Ltd., Ilford, United Kingdom).
11. 96-well white plate, polystyrene, High Bind, white flat-bottom wells, non-sterile, white (catalog number: CLS3922; Sigma-Aldrich, San Luis, MO, United States, Corning^®^).
12. 96-well black plate, polystyrene, flat bottom, black polystyrene, matrix active group High Bind, non-sterile (catalog number: CLS3925; Sigma-Aldrich, San Luis, MO, United States, Corning^®^).
13. 4-Morpholinepropanesulfonic (MOPS) acid (catalog number: M1254; CAS number: 1132-61-2; Sigma-Aldrich, San Luis, MO, United States); stored at room temperature (RT).
14. Adenosine 5′-diphosphate monopotassium salt dihydrate (ADP, catalog number: A5285, CAS number: 72696-48-1: Sigma-Aldrich, San Luis, MO, United States); stored at −20°C.
15. Adenosine 5′-triphosphate (ATP) assay mix (catalog number: FLAAM; Sigma-Aldrich, San Luis, MO, United States) stored at −20°C.
16. Amplex™ Red Reagent (catalog number: A12222; Thermo Fisher Scientific, Waltham, MA, United States) stored at −20°C.
17. Cyclosporin A (CsA, catalog number: 30024, CAS number: 59865-13-3; Sigma-Aldrich, San Luis, MO, United States) stored at −20°C.
18. Ethylene-bis(oxyethylene dinitrilo)tetraacetic acid (EGTA, catalog number: E0396, CAS number: 67-42-5; Sigma-Aldrich, San Luis, MO, United States) stored at RT.
19. Glutamic acid potassium (glutamate, catalog number: G1501, CAS number: 6382-01-0; Sigma-Aldrich, San Luis, MO, United States) stored at RT.
20. Bovine serum albumin (BSA, catalog number: A6003, CAS number: 9048-46-8; Sigma-Aldrich, San Luis, MO, United States) stored at 4°C. Critical: BSA is used to remove (bind) free fatty acids; therefore, use BSA-free fatty acids.
21. Calcium chloride dihydrate (CaCl_2_, catalog number: C3306, CAS number: 10035-04-8; Sigma-Aldrich, San Luis, MO, United States) stored at RT.
22. Carbonyl cyanide 4-(trifluoromethoxy)phenylhydrazone (FCCP, catalog number: C2920, CAS number: 370-86-5; Sigma-Aldrich, San Luis, MO, United States) stored at 4°C.
23. L-Ascorbic acid (ascorbate, catalog number: A5960, CAS number: 50-81-7; Sigma-Aldrich, San Luis, MO, United States) stored at 4°C.
24. Magnesium chloride hexahydrate (MgCl_2_, catalog number: M2393, CAS number: 7791-18-6; Sigma-Aldrich, San Luis, MO, United States) stored at 4°C.
25. L-(-)-Malic acid (malate, catalog number: M1000; CAS number: 97-67-6; Sigma-Aldrich, San Luis, MO, United States) stored at RT.
26. N-(2-Hydroxyethyl)piperazin-N′-(2-ethanesulfonic acid)] (HEPES, catalog number: H7006; CAS number: 75277-39-3; Sigma-Aldrich, San Luis, MO, United States) stored at RT.
27. N,N,N,N-Tetramethyl-p-phenylenediamine dihydrochloride (TMPD, catalog number: T739; Sigma-Aldrich, San Luis, MO, United States) stored at RT.
28. Potassium chloride (KCl, catalog number: P5405, CAS number: 7447-40-7; Sigma-Aldrich, San Luis, MO, United States) stored at RT.
29. Potassium dihydrogen phosphate (KH_2_PO_4_, catalog number: P5655, CAS number: 7778-77-0; Sigma-Aldrich, San Luis, MO, United States) stored at RT.
30. Sodium phosphate dibasic (Na_2_HPO_4_, catalog number: S3264, CAS number: 7558-79-4; Sigma-Aldrich, San Luis, MO, United States) stored at RT.
31. Succinic acid (succinate, catalog number: S3674, CAS number: 110-15-6; Sigma-Aldrich, San Luis, MO, United States) stored at RT.
32. Sucrose (catalog number: S7903, CAS number: 57-50-1; Sigma-Aldrich, San Luis, MO, United States) stored at RT.
33. Trizma base (Tris, catalog number: T6066, CAS number: 77-86-1; Sigma-Aldrich, San Luis, MO, United States) stored at RT.
34. Tetramethylrhodamine methyl ester perchlorate (TMRM, catalog number: T5428, CAS number: 115532-50-8; Sigma-Aldrich, San Luis, MO, United States) stored at −20°C.

### Recipes

1. Isolation buffer in mmol/l: sucrose 250; HEPES 10; EGTA 1, pH 7.4. Dissolve 85.58 g of sucrose, 2.6 g of HEPES, and
0.38 g of EGTA in 800 ml of ultrapure water. Adjust pH to 7.4 using 2 mol/l Tris, and bring the solution to 1 L and store at 4°C.
2. BSA isolation buffer: Dissolve 400 mg of BSA fat-free in 50 ml isolation buffer.
3. Electrolyte solution in mmol/l: Na_2_HPO_4_ 374; KH_2_PO_4_ 191; KCl 139.5; NaN_3_ 15.38. Dissolve 2.655 g of Na_2_HPO_4_, 1.3 g of KH_2_PO_4_, and 0.52 g of KCl in 50 ml ultrapure water. Add 0.05 g of NaN_3_ and a few crystals of AgCl to provide a saturated solution. Filtrate the solution and store at 4°C. Caution: NaN_3_ is highly toxic.
4. Incubation buffer for respiration pyruvate/malate (IBRP/M) in mmol/l: 125 KCl; 10 MOPS; 5 MgCl_2_; 5 KH_2_PO_4_;0.02 EGTA; 5 pyruvate/malate, pH 7.4. Add 6.25 ml of 1 mol/l KCl, 1 ml of 500 mmol/l MOPS, 0.1 ml of 1 mol/l MgCl_2_, 0.25 ml of 1 mol/l KH_2_PO_4_, 0.1 ml of 100 mmol/l EGTA, and 1 ml of 250/250 mol/l pyruvate/malate. Adjust pH to 7.4 using 500 mmol/l Tris, and bring the solution to 50 ml using ultrapure water and filtrate it. Store at 4°C.
5. Incubation buffer for respiration succinate (IBRS) in mmol/l: 125 KCl; 10 MOPS; 5 MgCl_2_; 5 KH_2_PO_4_;0.02 EGTA; 5 succinate, pH 7.4. Add 6.25 ml of 1 mol/l KCl, 1 ml of 500 mmol/l MOPS, 0.1 ml of 1 mol/l MgCl_2_, 0.25 ml of 1 mol/l KH_2_PO_4_, 0.1 ml of 100 mmol/l EGTA, and 1 ml of 250 mmol/l succinate. Adjust pH to 7.4 using 0.5 mol/l Tris, and bring the solution to 50 ml using ultrapure water, and filtrate it and store at 4°C.
6. 100 mmol/l ADP: Dissolve 427 mg of ADP in 10 ml of ultrapure water. Prepare 100 μl aliquots and store at −20°C.
7. 500 mmol/l ascorbate: Dissolve 880.65 mg of ascorbic acid in 10 ml of ultrapure water. Prepare 100 μl aliquots and store at −20°C.
8. 10 mmol/l calcium chloride: Dissolve 55.49 mg of CaCl_2_ in 50 ml of ultrapure water and store at −20°C.
9. 10 mmol/l cyclosporin A: Dissolve 12 mg of cyclosporin A in 1 ml of absolute ethanol and store at −20°C.
10. 0.1 mol/l EGTA stock solution: Dissolve 1.9 g of EGTA in 30 ml of ultrapure water. Adjust pH to 7.4 using 0.5 mol/l Tris and dilute to 50 ml. Store at 4°C.
11. 10 mmol/l FCCP stock solution: Dissolve 2.5 mg of FCCP in 1 ml of absolute ethanol. Store at −20°C. Dilute the stock solution to 5 μM by adding 5 μl of 10 mmol/l FCCP in 10 ml of absolute ethanol. Prepare 20 μl aliquots and store at −20°C.
12. 0.25 mol/l Pyruvate/0.25 mol/l malate stock solution: Dissolve 1.38 g of pyruvate and 1.68 g of malate in 30 ml of ultrapure water and adjust pH to 7.4 with 2 mol/l Tris. Dilute to 50 ml and store at 4°C.
13. 1 mol/l KCl stock solution: Dissolve 18.64 g of KCl in 250 ml of ultrapure water and store at 4°C.
14. 1 mol/l KH_2_PO_4_ stock solution: Dissolve 6.8 g of KH_2_PO_4_ in 30 ml of ultrapure water. Adjust pH to 7.4 using 0.5 mol/l Tris and dilute to 50 ml. Store at 4°C.
15. 1 mol/l MgCl_2_ stock solution: Dissolve 4.7 g of MgCl_2_ in 50 ml of ultrapure water and store at 4°C.
16. 0.5 mol/l MOPS stock solution: Dissolve 10.46 g of MOPS in 30 ml of ultrapure water. Adjust pH to 7.4 using 0.5 mol/l Tris and dilute to 100 ml. Store at 4°C.
17. 1 mmol/l rotenone stock solution: Dissolve 3.9 mg of rotenone in 10 ml of absolute ethanol. Dilute the stock solution to 500 μmol/l by adding 5 ml of 1 mM rotenone in 5 ml of absolute ethanol. Prepare 200-μl aliquots and store at −20°C (critical step). Rotenone is light-sensitive. The stock solution should be protected from direct light.
18. 0.25 mol/l succinate stock solution: Dissolve 2.02 g of succinate in 30 ml of ultrapure water and adjust pH to 7.4 with 2 mol/l Tris. Dilute to 50 ml and store at 4°C.
19. 150 mmol/l TMPD: Dissolve 49.3 mg of TMPD in 2 ml DMSO. Prepare 10-μl aliquots and store at −20°C.
20. 2 mol/l Tris: Dissolve 121.14 g of Tris in 500 ml of ultrapure water. Dilute to 0.5 mol/l by adding 250 ml 2 mol/l Tris in 750 ml ultrapure water and store at RT.
21. 5 mmol/l TMRM stock solution: Dissolve 5 mg of TMRM in 2 ml of DMSO. Store at −20°C.

### Equipment

1. Surgery scissors (ABC *instrumentos cirúrgicos*, surgery scissors straight 12 cm, code: 321. Catalog number: 10304850053).
2. Refrigerated highest-speed centrifuge (Mikro 200R; Hettich, Tuttlingen, Germany).
3. Tissue homogenizer (T 25 Digital ULTRA-TURRAX^®^, catalog number: 3725000; Merck, Darmstadt, Germany).
4. Clark-type oxygen electrode and respirometer MT200A (oxygen meter, 782, MT200A; Strathkelvin, Motherwell, Scotland).
5. Spectrofluorimeter SpectraMax^®^ M3 (SpectraMax^®^ M3; Molecular Devices, San Jose, CA, Untied States).
6. Centrifuges and rotors: Precool centrifuges and rotors to 4°C.
7. Oxygraph chamber: Adjust the temperature of the water bath to 37°C. Calibrate the Clarke-type oxygen electrode. Procedures may vary from instrument to instrument. Follow the manual for the oxygen electrode and chamber you are using (Strathkelvin 782 2-channel Oxygen System version 1.0; Strathkelvin, Motherwell, Scotland).
8. Spectrophotometer: Adjust the temperature of the cuvette block to 37°C.

### Software

1. Strathkelvin 782 2-channel Oxygen System version 1.0 (Oxygenmeter, 782; Strathkelvin, Motherwell, Scotland).
2. SoftMax^®^ Pro Software (Molecular Devices, San Jose, CA, United States).
3. GraphPad Prism 8.4.3 (San Diego, CA, United States).

## Methods

### Animals

CD-1 mice (25–30 g BW) were used. The animal study was reviewed and approved by our institutional ethics committee on the use of animals (Health Sciences Center, Federal University of Rio de Janeiro (protocol 015/17) and followed the guidelines of the Brazilian National Council for Animal Experimentation Control, Ministry of Science, Technology, and Innovation (CONCEA/MCTI), and the Guide for the Care and Use of Laboratory Animals published by the United States National Institutes of Health (8th edition, 2011).

### Isolation of Mitochondria

The experimental protocol must be available after lung mitochondrial isolation, because mitochondria are viable for about 4 h only (critical step).

#### Collection of Tissue Samples (Timing Is 2–5 Min Per Animal)

The mice were euthanized and underwent a bilateral thoracotomy. The lungs were carefully removed *en bloc* and immediately placed in a tube containing an ice isolation buffer at 4°C (see section “Recipes”) to remove excess blood.

#### Isolation of Mitochondria (Timing Is Approximately 40–90 Min)

**FIGURE 1:**
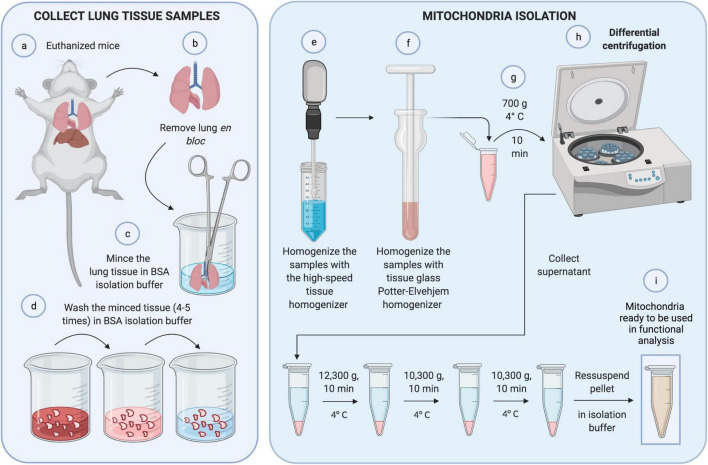
Mitochondrial isolation. First, **(a,b)** lungs were removed *in bloc* from euthanized mice. **(c)** Lung tissue was minced with scissors and **(d)** washed with BSA isolation buffer four to five times to eliminate blood. **(e)** The sample was homogenized with a high-speed tissue homogenizer and, again, with tissue glass Potter-Elvehjem homogenizer carefully, to achieve cell disruption without compromising mitochondrial integrity **(f)**. **(g)** The homogenate was centrifuged at 700 g for 10 min at 4°C. **(h)** The pellet was discarded, and the supernatant was collected and submitted to differential centrifugation. Finally, **(i)** the resultant pellet containing mitochondria was ready to be used in functional analyses.

The following steps are critical for the isolation of mitochondria (critical step). Mistakes during mitochondria isolation are irreversible and can spoil the running experiment. All processes must be performed on ice. Centrifugation steps at 4°C and buffers should be precooled during processing. It is important to work fast to avoid delays in tissue preparation (Figure 1).

a. Remove the adipose tissue and all large vessels using scissors.
b. Mince the tissue into 1–2 g fragments and transfer each one into a Teflon beaker with 10 ml BSA isolation buffer on ice.
c. Remove all remaining fat. The tissue must be thoroughly minced, since the size of the sample directly affects the subsequent homogenization step and eventually the yield of mitochondria (critical step). Ensure the removal of all fats that also affect the yield of mitochondria.
d. Split the minced tissue from one Teflon beaker into two 14-ml round-bottom tubes. The tube should not contain more than 2 ml tissue volume. Whenever necessary, use more 14-ml round-bottom tubes.
e. Wash the minced tissue samples: fill the 14-ml round-bottom tubes with 10 ml BSA isolation buffer, let the tissue sink, remove the buffer, and repeat tissue washing until the buffer is clear (the minced tissue would then contain no blood). Usually, four or five washings are enough to obtain a clean BSA isolation buffer. Hence, fill the 14-ml round-bottom tubes up to 6 ml with isolation buffer. The optimal tissue/buffer ratio is 1:3 or less. After mincing the tissue, part of it precipitates and some pieces float because of the air in the air spaces (critical step). Be careful in removing the blood during washing to avoid tissue loss.
f. Homogenize the samples with the tissue homogenizer (Ultra-Turrax) using two 10-s treatments at a shaft rotation rate of 6,500 × g each. Perform the homogenization on ice with slight movements of the centrifuge tube. Wait for 10 s between the homogenization steps to avoid heating of the homogenizer and the samples, and to avoid foaming (critical step).
g. Collect the samples and transfer them to a tissue glass Potter-Elvehjem homogenizer. Homogenize the samples, and stroke the suspension about 30–40 times. This procedure can compromise mitochondrial integrity if not done carefully (critical step). It Is recommended to precool the glassware in an ice bath 5–10 min before starting the procedure. Attention: The use of proteases, e.g., nargase, during mitochondrial isolation, commonly performed in other tissues such as the heart, ruins the whole process.
h. Centrifuge the homogenate at 700 × g for 10 min at 4°C.
i. Collect the supernatant in 2-ml microfuge tubes and discard the pellets. Centrifuge the supernatant at 12,300 × g for 10 min at 4°C.
j. Discard the supernatant and resuspend the pellet in 0.5 ml of ice-cold isolation buffer by gentle pipetting, and collect the mitochondrial suspensions in 2-ml microfuge tubes. Avoid the formation of foam during the resuspension process (critical step).
k. Centrifuge the supernatant in ice-cold isolation buffer at 10,300 × g for 10 min at 4°C.
l. Pool all the mitochondrial suspensions in one 2-ml microfuge tube and repeat the previous step.
m. Resuspend the resulting pellet in 100–200 μl isolation buffer and store it on ice. Resuspend the pellets carefully by gentle pipetting to obtain a uniform suspension without any visible clump (critical step).
n. Measure mitochondrial concentration using the Lowry or BSA method.

#### Pause Point

At this point, the mitochondria are ready to be used in experiments to explore their function. Use the preparation within 4 h for better functional responses. Store the mitochondrial suspension on ice.

Note: The isolated mitochondria by this protocol can be used in different oximeter equipment, and can be analyzed with different software and methodologies.

### Mitochondrial Oxygen Consumption (Timing: Approximately 10–20 Min Per Measurement)

In each experiment, use 200 μg of protein per ml for good acquisition data. The oxygen consumption gives information about the electron transport chain and the oxidative phosphorylation of the mitochondria. By the addition of substrates and inhibitors, or by uncoupling oxidative phosphorylation, it is possible to modulate the rate of oxygen consumption and gain further insight into the activity of each complex of the electron transport chain.

**FIGURE 2:**
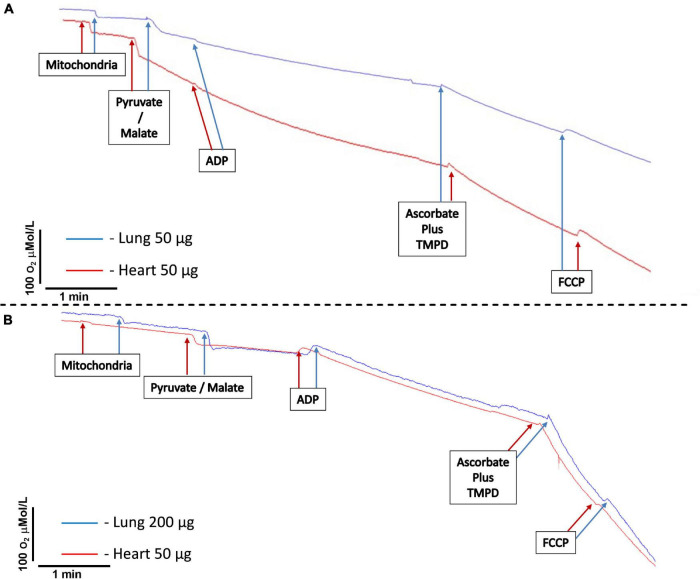
Representative tracings of mitochondria oxygen consumption. **(A)** Comparison between isolated mitochondria loading from heart (50 μg) and lung (50 μg) tissues. **(B)** Comparison between isolated mitochondria loading from heart (50 μg) and lung (200 μg) tissues. Mitochondria represents the moment of the addition of isolated mitochondria. Pyruvate/malate represents the time of addition of pyruvate 5 μmol/L/malate 5 μmol/L. ADP signals the time to add ADP 400 μmol/L. Ascorbate 3 mmol/L plus N,N,N,N-tetramethyl-p-phenylenediamine dihydrochloride (TMPD) 300 μmol/L indicates the addition of ascorbate and TMPD. FCCP indicates the addition of carbonyl cyanide 4-(trifluoromethoxy)phenylhydrazone 30 nmol/L.

At this point, mitochondrial complexes I (states 1, 2, and 3), II (state 3), and IV respiration with subsequent uncoupling of oxidative phosphorylation were measured in a two-chamber respirometer. With two different chambers, it is possible to measure two different experimental groups at the same time, observing results in mitochondrial function in parallel (critical step). Moreover, the respiration of complex I and complex II can be available in parallel using two different chambers.

a. Add 0.5 ml of IBRP/M buffer to the chambers. One can opt to add IBRP/M without pyruvate/malate (or 5 mmol/l glutamate/5 mmol/l malate, bypassing the critical step of pyruvate decarboxylation, which is highly dependent on NAD^+^) to measure the state 1 respiration of complex I and to add IBRS plus 2 μmol/l rotenone to the other chamber to measure complex II respiration. Make sure that the magnetic stirrer moves constantly. Rotenone is sticky and inhibits complex I respiration (critical step). Therefore, we suggest washing each chamber that received rotenone with a cardiac or liver tissue homogenate to assist the removal of rotenone. Additionally, we recommend washing the chamber three times with 70° alcohol, followed by three washes with EDTA 100 mmol/l. Finally, wash 10 times with MilliQ water.
b. Equilibrate the temperature and oxygen tension of the buffer and close the chamber. Usually, 3–4 min are sufficient until the oxygen concentration in the chamber remains stable.
c. Start the recording of the oxygen concentration in the chamber. Steady-state recording without drifts is mandatory (critical step). Wait for 1–5 min to obtain a stable baseline. A maximal drift of ±10 nmol O_2_/min is considered acceptable once endogenous substrates could be present in the preparation starting State 2 respiration before the addition of the exogenous substrate. Add 200 μg of mitochondrial protein using a Hamilton syringe and record for 3 min. If one chooses to add IBRP/M without pyruvate/malate, the state 1 of complex I is measured. One should add pyruvate/malate 5 μmol/l, and the oxygen concentration in the chamber will decrease because of oxygen consumption by the mitochondria, which can be referred to as state 2 complex 1 respiration. and record for 3 min (Figure 2).
d. Add 4 μl of 100 mmol/l ADP to obtain a final concentration of 400 μmol/l using a Hamilton syringe and record oxygen concentration for 3 min (Figure 2).
e. The decrease in oxygen concentration speeds up, caused by stimulating mitochondrial respiration with ADP (state 3). ADP-stimulated respiration should be faster than baseline respiration, reflecting good coupling of mitochondria (critical step). The respiration could slow down and return to a rate comparable to that of the baseline respiratory state, as result of the conversion of all added ADP and phosphate into ATP.
f. Add simultaneously 2 μl of 150 mmol/l TMPD and 6 μl of 500 mmol/l ascorbate to obtain final concentrations of 300 and 3 mmol/l, respectively. Record oxygen concentration for 1 min. The oxygen concentration will decrease faster than with ADP stimulation. TMPD is an electron donor to complex VI, which is readily reduced by ascorbate and oxidized by cytochrome C (Figure 2).
g. Add 6 μl of 5 μmol/l FCCP to obtain a final concentration of 30 nmol/l. Record oxygen concentration for 1 min. The oxygen concentration will decrease further. FCCP is an uncoupling agent, which turns the mitochondrial membrane permeable to protons and, therefore, eliminates the chemiosmotic gradient. As a result, ATP synthesis is disrupted (Figure 2).
h. Stop recording.
i. Calculate mitochondrial oxygen consumption using the software Analysis (Strathkelvin 782 2-channel Oxygen System version 1.0; Strathkelvin, North Lanarkshire, Scotland) or similar. Calculate baseline oxygen consumption 75 s after the addition of mitochondria. Calculate state 2 complex I oxygen consumption 75 s after the addition of pyruvate/malate. Determine ADP-stimulated respiration 30 s after the addition of ADP. Determine complex VI respiration 30 s after the addition of ascorbate/TMPD. Calculate maximal uncoupled respiration rate 30 s after the addition of FCCP.

### Measurement of Adenosine 5′-Triphosphate Production (Timing: Approximately 20 Min)

In the respiration chamber, repeat all the steps up to adding 4 μl of 100 mmol/l ADP to obtain a final concentration of 400 μmol/l. Then, record the ADP-stimulated respiration for 3 min. Thereafter, the incubation buffer containing mitochondria should be transferred to an Eppendorf tube and immediately supplemented with ATP assay mix (ATP Bioluminescence Assay Kit; Sigma-Aldrich, St. Louis, MO, United States) diluted to 1:5 (incubation buffer containing mitochondria: ATP assay mix). Mitochondrial ATP production was determined immediately after each respiration measurement and compared with ATP standards using a 96-well white plate in a spectrofluorometer (SpectraMax^®^ M3; Molecular Devices, San Jose, CA, United States) at 560-nm emission.

### Measurement of Mitochondrial ROS (Timing: Approximately 30 Min)

The Amplex Red Hydrogen Peroxide Assay (catalog number: A12222; Thermo Fisher Scientific, Waltham, MA, United States) was used to determine mitochondrial ROS concentration. Amplex Red reacts in 1:1 stoichiometry with peroxide in the presence of horseradish peroxidase (HRP) and produces highly fluorescent 95% resorufin. The incubation buffer containing mitochondria should be transferred to an Eppendorf tube and immediately supplemented with 50 μmol/l Amplex UltraRed Reagent (Thermo Fisher Scientific, Waltham, MA, United States) and 2 U/ml Pierce™ horseradish peroxidase (HRP, catalog number: 31491; Thermo Fisher Scientific, Waltham, MA, United States). The supernatant was collected after 20 min of incubation in the dark. Mitochondrial ROS concentration was determined and compared with H_2_O_2_ standards using a 96-well black plate and a spectrofluorometer (SpectraMax^®^ M3; Molecular Devices, San Jose, CA, United States) at 540-nm emission and 580-nm extinction (Maciel et al., 2020).

### Measurement of Mitochondrial Swelling

The integrity of the mitochondrial membrane was assessed by osmotically induced volume changes of the mitochondria and spectrophotometric determination of the apparent absorption of the suspension at 540 nm. A mitochondrial suspension (200 mg/ml) was added to the respiration medium in the absence of respiratory substrates, at 37°C, and under constant stirring. Mitochondrial swelling was stimulated with 1 μl of calcium chloride at 20 μmol/l to reach 100 nmol/l in 200 μl of mitochondrial suspension. Swelling was expressed as percentage of the absorption of the solution containing mitochondria in the presence of cyclosporin A 10 μmol/l (mitochondrial swelling = 0%) in relation to that absorbed after the addition of FCCP 1 μmol/l (mitochondrial swelling = 100%).

### Measurement of Mitochondrial Transmembrane Potential (ΔΨm)

For ΔΨm determination, the probe tetramethylrhodamine methyl ester (TMRM, 400 nmol/l) was added to the respiration solution containing 200 mg/ml of mitochondria and incubated for 1 h at 4°C before the experiment. ΔΨm was estimated by the fluorescence emitted by TMRM under 580-nm excitation. ΔΨm was expressed as the percentage of fluorescence emitted by TMRM-labeled mitochondria in the presence of cyclosporin A (mitochondrial despolarization = 0%), relative to that emitted after the addition of FCCP to fully depolarize the mitochondria (mitochondrial despolarization = 100%).

### Electron Leakage and ATP/ROS Production Ratio

Electron leakage is the loss of the electron from the electron transport chain to form superoxide (O_2_-). However, other reactive oxygen species, such as hydroperoxyl radical (HO_2_) and hydrogen peroxide (H_2_O_2_), might occur spontaneously (e.g., pH-dependent) or under the action of antioxidant enzymes (e.g., superoxide dismutase). The site of initial leakage is often considered to be a semiquinone radical (QH) or reduced flavin (FMN and FAD) (A–B). To calculate the fraction of electrons that leaked out of the respiratory chain, the rate of H_2_O_2_ formation (see section E) is divided by the rate of mitochondrial O_2_ consumption (see section C). H_2_O_2_ production and oxygen consumption rates must be expressed using the same units and correspond to the same respiratory state (C–E). The ATP/ROS reason should be measured to determine the formation of ROS linked to O_2_ consumption. Thus, we were able to determine the electron leakage inherent to ROS production (Santiago et al., 2008; Murphy, 2009; Jastroch et al., 2010; Daussin et al., 2012).

### Statistical Analysis

Three experimental groups were tested. The first one corresponded to isolated mitochondria from hearts with a protein load of 50 μg in each experiment. The second group consisted of isolated mitochondria from lungs with a protein load of 50 μg in each experiment. The third group contained mitochondria isolated from lungs with a protein load of 200 μg in each experiment. For graphic and statistical analysis, the software GraphPad Prism 8.4.3 (San Diego, CA, United States) was used. The significance of observed differences in mitochondrial oxygen consumption and functions was evaluated by the parametric one-Way ANOVA test followed by Tukey’s multiple comparisons test. In all cases, *p* < 0.05 was considered to be significant. Experimental values are reported as mean ± standard deviation.

## Results

The differences between the present protocol and previous protocols are shown in Table 1.

**TABLE 1:** Differences between the present protocol and previous protocols.

Present protocol	Spear and Lumeng, 1978	Zhang et al. (2018)	Gedik et al., 2017	Maciel et al., 2020
BSA fat-free **0.8%**	BSA fat-free **0.5%**	BSA fat-free **2%** and computational model	BSA fat-free **0.5%**	BSA fat-free **0.1%**
Remove blood content from the tissue **without** losing large amounts of sample.	Remove blood content from the tissue losing large amounts of sample	Remove blood content from the tissue losing large amounts of sample and computational model	Remove blood content from the tissue losing large amounts of sample	Remove blood content from the tissue losing large amounts of sample
Mitochondria concentration **200 μ g/ml**	**No information**	Mitochondria concentration **1 mg/ml** computational model	Mitochondria concentration **50 μ g/ml**	Mitochondria concentration **50 μ g/ml**
**No** use of proteases	**No information**	Protease inhibitor cocktail Set III	Use of proteases (nargase)	Use of proteases (nargase)
**Lung** tissue	Lung tissue	Lung mitocôndria, and computational model	Heart tissue	Heart tissue
**Enough** material to grant the completion of several experiments	**No** Enough material to grant the completion of several experiments	**No** Enough material to grant the completion of several experiments, computational model	Enough material to grant the completion of several experiments	Enough material to grant the completion of several experiments
**Mice**	Rat, rabbits, and mice	Rat and computational model	Rat	Rat

*The bold terms highlight the difference between the protocols.*

### Mitochondrial Respiration

**FIGURE 3:**
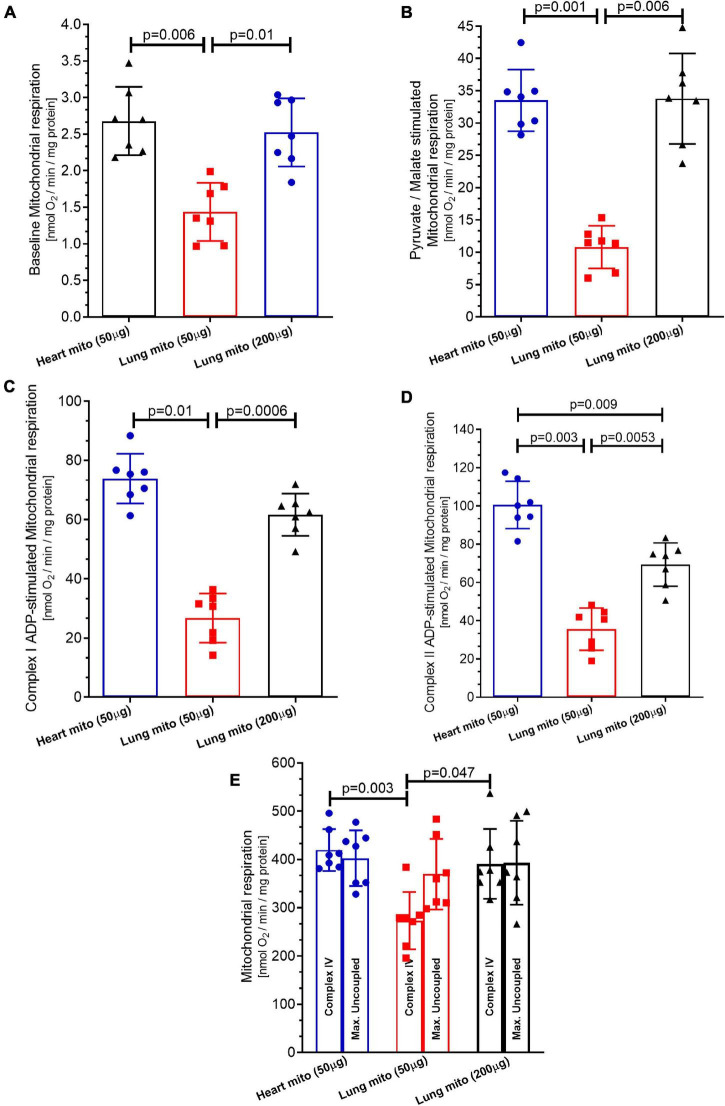
Mitochondrial respiration. **(A)** Baseline respiration (state 1 complex 1), **(B)** pyruvate/malate stimulation (state 2 complex 1) respiration, **(C)** adenosine diphosphate (ADP) stimulation (state 3 complex 1) respiration. **(D)** Complex II respiration was stimulated with succinate and using the complex I inhibitor Rotenone. **(E)** Complex IV respiration stimulated with N,N,N,N-tetramethyl-p-phenylenediamine dihydrochloride (TMPD) and ascorbate and maximal uncoupled oxygen uptake induced by carbonyl cyanide 4-(trifluoromethoxy)phenylhydrazone (FCCP) of isolated mitochondria from mice heart and lung. Heart mito (50 μg) represents the group of isolated mitochondria from hearts. Lung mito (50 μg) indicates the group of isolated mitochondria from lungs with loading of 50 μg. Lung mito (200 μg) signals the group of isolated mitochondria from lungs with loading of 200 μg. Each symbol represents one animal. The values are reported as mean ± standard deviation. Horizontal square brackets indicate significantly different differences and the corresponding *p*-value.

Figure 3A depicts that the mitochondrial oxygen consumption by complex I under state 1 was smaller in lung mitochondria-50 μg (1.43 ± 0.39 nmol O_2_/min/mg protein) than in heart mitochondria (2.68 ± 0.46 nmol O_2_/min/mg protein, *p* = 0.006) and in lung mitochondria-200 μg (2.52 ± 0.45 nmol O_2_/min/mg protein, *p* = 0.01), which did not differ between them (*p* = 0.7).

As shown in Figure 3B, the mitochondrial oxygen consumption by Complex I under state 2 is smaller in lung mitochondria-50 μg (10.79 ± 3.3 nmol O_2_/min/mg protein) than in heart mitochondria (33.51 ± 4.7 nmol O_2_/min/mg protein, *p* = 0.001) and in lung mitochondria-200 μg (33.78 ± 7 nmol O_2_/min/mg protein, *p* = 0.006), which did not differ between them (*p* = 0.99).

The mitochondrial oxygen consumption by complex I under state 3 was smaller in lung mitochondria-50 μg (26.75 ± 8.2 nmol O_2_/min/mg protein) than in heart mitochondria (73.83 ± 8.38 nmol O_2_/min/mg protein, *p* = 0.01) and in lung mitochondria-200 μg (61.67 ± 9.1 nmol O_2_/min/mg protein, *p* = 0.0006), which did not differ, *p* = 0.99, as presented in Figure 3C.

The mitochondrial oxygen consumption by complex II under state 3 was smaller in lung mitochondria-50 μg (35.54 ± 11 nmol O_2_/min/mg protein) than in heart mitochondria-50 μg (100.5 ± 12.4 nmol O_2_/min/mg protein, respectively, *p* = 0.003), and in lung mitochondria-200 μg (69.32 ± 11.3 nmol O_2_/min/mg protein, *p* = 0.0053). However, the increase in oxygen consumption by lung mitochondria-200 μg did not reach the level of the heart mitochondria-50 μg (*p* = 0.009), as shown in Figure 3D.

Figure 3E shows that the mitochondrial oxygen consumption by complex IV was smaller in lung mitochondria-50 μg (273.3 ± 59 nmol O_2_/min/mg protein) than in heart mitochondria-50 μg (419.5 ± 43 nmol O_2_/min/mg protein, *p* = 0.003) and lung mitochondria-200 μg (392.1 ± 72.4 nmol O_2_/min/mg protein, *p* = 0.047), which did not differ between them (*p* = 0.19). The mitochondrial oxygen consumption by maximal oxygen uptake of uncoupled mitochondria was similar in heart mitochondria-50 μg and lung mitochondria-50 μg (402.5 ± 57.5 and 369.51 ± 72.8 nmol O_2_/min/mg protein, respectively, *p* = 0.7). Lung mitochondria-200 μg showed similar respiration to heart mitochondria-50 μg (393.45 ± 86.12 nmol O_2_/min/mg protein, *p* = 0.17 vs. heart mitochondria-50 μg) and *p* = 0.5 vs. lung mitochondria-50 μg) (Figure 3E).

### Mitochondrial ROS Production

**FIGURE 4:**
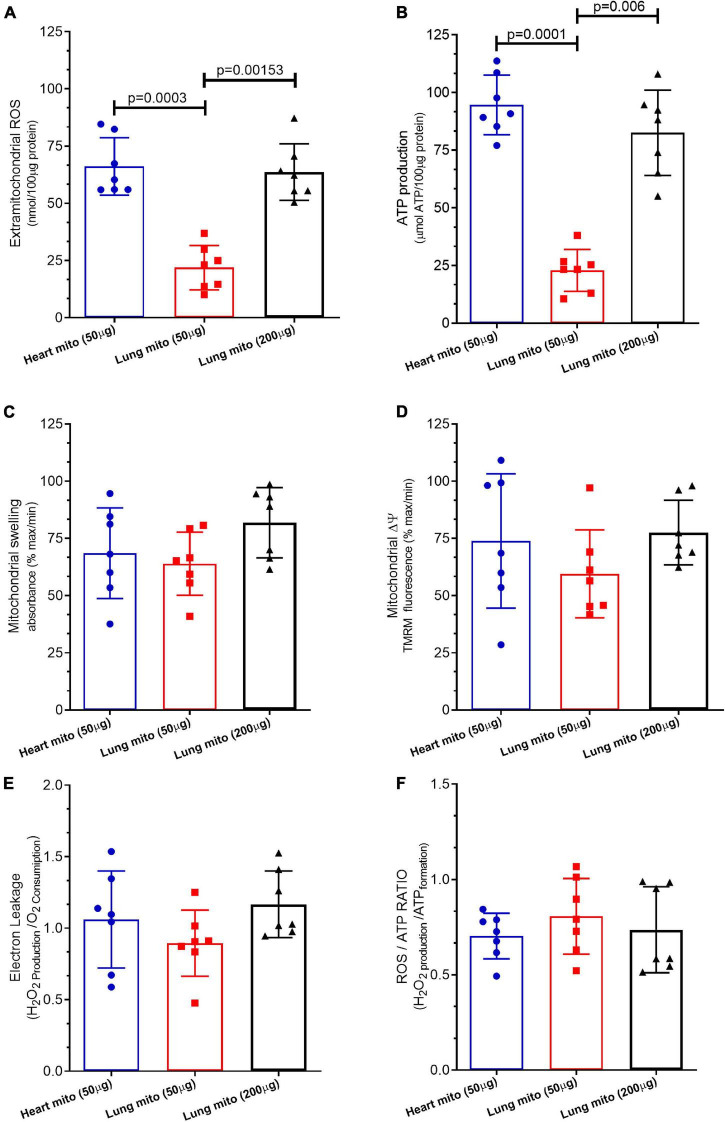
Mitochondrial products and characteristics. **(A)** Reactive oxygen species (ROS) production, **(B)** adenosine triphosphate (ATP) production, **(C)** mitochondrial swelling, **(D)** mitochondrial transmembrane potential (mΔψ), **(E)** electron leakage, and **(F)** ROS/ATP ratio of isolated mitochondria from mice heart and lung. Heart mito (50 μg) represents the group of isolated mitochondria from hearts. Lung mito (50 μg) indicates the group of isolated mitochondria from lungs with loading of 50 μg. Lung mito (200 μg) signals the group of isolated mitochondria from lungs with loading of 200 μg. Each symbol represents one animal. Data are expressed as mean ± standard deviation. Horizontal square brackets indicate significantly different differences and the corresponding *p*-value.

Mitochondrial ROS production by lung mitochondria-50 μg (21.87 ± 9.6 nmol/loaded protein) was lower than that by heart mitochondria-50 μg (66.08 ± 12.5 nmol/loaded protein, *p* = 0.003) and lung mitochondria-200 μg (63.64 ± 12.3 nmol/loaded μg protein, *p* = 0.0015), which was similar (*p* = 0.87), as displayed in Figure 4A.

### Mitochondrial Adenosine 5′-Triphosphate Production

Figure 4B shows that mitochondrial the ATP production by lung mitochondria-50 μg (22.92 ± 9.12 μmol ATP/loaded protein) was lower than that by heart mitochondria-50 μg (91.62 ± 5.9 μmol ATP/loaded protein, *p* = 0.0001) and lung mitochondria-200 μg (83.51 ± 9.49 μmol ATP/loaded protein, *p* = 0.006), which was similar (*p* = 0.075).

### Mitochondrial Swelling

The mitochondrial swelling of lung mitochondria-50 μg (68.5 ± 19.7% maximum), heart mitochondria-50 μg (63.9 ± 13.8% maximum), and lung mitochondria-200 μg (81.84 ± 15.35% maximum) did not differ among them (*p* = 0.3), as depicted in Figure 4C.

### Mitochondrial Δψ

The mitochondrial Δψm of lung mitochondria-50 μg (73.84 ± 29.3% maximum), heart mitochondria-50 μg (59.46 ± 19.2% maximum), and lung mitochondria-200 μg (77.51 ± 14.1% maximum) did not differ among them (*p* = 0.52) (Figure 4D).

### Mitochondrial Proton Leakage

Figure 4E shows that the mitochondrial proton leakage by lung mitochondria-50 μg (1.04 ± 0.39 H_2_O_2_ production/O_2_ consumption), heart mitochondria-50 μg (0.84 ± 0.31 H_2_O_2_ production/O_2_ consumption), and lung mitochondria-200 μg (0.97 ± 0.22 H_2_O_2_ production/O_2_ consumption) was similar (*p* = 0.48).

### Mitochondrial ATP/ROS Ratio

The mitochondrial ATP/ROS ratio of lung mitochondria-50 μg (1.26 ± 0.19 H_2_O_2_ production/ATP formation), heart mitochondria-50 μg (1.07 ± 0.42 H_2_O_2_ production/ATP formation), and lung mitochondria-200 μg (1.39 ± 0.35 H_2_O_2_ production/ATP formation) did not differ (*p* = 0.54), as depicted in Figure 4F.

## Discussion

We used herein a new protocol with specific and detailed steps aiming to improve mitochondrial isolation from lung tissue. It was abridgedly published (Caldeira et al., 2021) but not tested against a well-documented and broadly used one (Gedik et al., 2017). This new protocol improves the acquisition of a robust and preserved sample of isolated mitochondria, allowing a range of analyses with the same sample, increasing mitochondria viability and experimental reproducibility. Here, we describe step-by-step the instructions for lung mitochondria isolation and warn for critical steps (steps of the procedure in which the researcher must be extremely careful, or attentive, with the procedure for the perfect execution of the isolation). Before our improved method, there was no consensus concerning protocols for mitochondrial isolation from the lung tissue (Zhang et al., 2018). The isolation of mitochondria from lung tissue is extremely difficult, because of the elevated fatty acid content and low load of mitochondria in pulmonary cells (Spear and Lumeng, 1978). Therefore, the isolation buffer most contain a high amount of fat-free BSA to be bound to free fatty acids. Noteworthy is that the amount of fat-free BSA used to isolate mitochondria from cardiomyocytes (Maciel et al., 2020) is half of that used to isolate mitochondria from the lung.

The integrity of the membrane in the lung-isolated mitochondria is of paramount importance (Zhang et al., 2018). Consequently, we detailed each step of our protocol very carefully. Because of lung intrinsic characteristic as an air-filled organ, there are difficulties in stages that aim to mince the tissue and remove the residual blood (Spear and Lumeng, 1978). The removal of blood (including hemoglobin) from tissue is mandatory to avoid oxygen sequestration during O_2_- consumption assay. This singular lung characteristic undermines the isolation of mitochondria, resulting in low success rate and small amount of mitochondria available for putatively several functional tests (Spear and Lumeng, 1978). These issues pertaining to an air-filled highly perfused organ were overcome by our improved method. Thus, we showed that it is possible to appropriately mince the lung and remove its blood content without losing large amounts of sample.

We compared mitochondrial functional characteristics using three experimental groups. The first one (control group) comprised mitochondria isolated from the heart carrying 50 μg of protein per experiment. Heart mitochondria are isolated by means of a well-established broadly used protocol (Schulz et al., 2015; Gedik et al., 2017; Maciel et al., 2020, 2021). The second experimental group contained isolated lung mitochondria carrying 50 μg of protein per experiment, the same loading as isolated heart mitochondria. The third experimental group consisted of isolated lung mitochondria at a concentration of 200 μg of protein per experiment. Mitochondrial respiration was measured with a Clark-type electrode at 37°C during magnetic stirring and consistently demonstrated that the concentration of lung-isolated mitochondria can affect the results. An identical concentration of lung- and cardiomyocyte-isolated mitochondria (50 μg) yielded smaller oxygen consumption by lung mitochondria at baseline respiration (Figures 2A, 3A), following pyruvate/malate (Figures 2A,B), ADP (Figures 2A, 3C), and TMPD/ascorbate (Figures 2A, 3D) titration. Taken together, these data strongly indicate that the loading of the lung mitochondria is not adequate using the 50 μg protein concentration. Protein dosage is an indirect measure of the concentration of mitochondria; therefore, a loading control that assesses functionality is necessary (Maciel et al., 2020). For such a purpose, the activation of mitochondrial complex IV is commonly employed as a loading control (Schulz et al., 2015; Gedik et al., 2017; Maciel et al., 2020, 2021), and we observed that the oxygen consumption in complex IV was less in isolated lung mitochondria loaded with 50 μg of protein than in the group of isolated heart mitochondria. Interestingly, heart isolated mitochondria presented similar values to those from lung isolated mitochondria loaded with 200 μg of protein (Figures 2B, 3). These data could suggest that lung tissue yields fewer mitochondria, and that greater loading is required to generate data comparable to those from heart tissue mitochondria. However, we cannot exclude the possibility that cardiac mitochondrion may have higher metabolism than lung mitochondria (Zhang et al., 2018; Spear and Lumeng; 1978). On the other hand, our FCCP-induced uncoupled respiration did not differ between all groups, suggesting that the mitochondria groups appear to have similar viability and behavior (Schulz et al., 2015; Gedik et al., 2017; Maciel et al., 2020, 2021). Indeed, 200 μg of protein per experiment is an acceptable amount, and our samples of isolated mitochondria had enough material to grant the completion of several experiments. Other techniques to analyze mitochondrial function, such as ATP production (Figure 4A) and ROS formation (Figure 4B), display the same trend. On the other hand, mitochondrial swelling (Figure 4C), mitochondrial transmembrane potential (Figure 4D), electron leakage (Figure 4E), and ROS/ATP ratio (Figure 4F) did not show a significant difference among the three groups, perhaps because mitochondrial swelling is analyzed by light scattering in the assay (Chapa-Dubocq et al., 2018). Mitochondrial transmembrane potential is analyzed by the stimulation of a fluorophore (Creed and McKenzie, 2019); and electron leakage and ROS/ATP ratio are calculated from existing data that were not challenged by experimental maneuvers (Santiago et al., 2008; Murphy, 2009; Jastroch et al., 2010; Daussin et al., 2012).

### Limitations

The lung is an extremely complex organ with regard to the heterogeneity of cells. Our method does not contemplate analyzing all 40 subtypes of cells found in the lung. However, this heterogeneity is an intrinsic part of the lung, and all methods of mitochondria isolation, for most diverse tissues, contemplate entire organ isolation, because different cells form a syncytium for the organ to work, e.g., the heart (Gedik et al., 2017), kidney (Schulz et al., 2015), liver (Goudarzi et al., 2018), adipose tissue (Matta et al., 2021), and brain (Marques Neto et al., 2020).

## Conclusion

Based on the method briefly described by Caldeira et al. (2021), we developed an optimized and successful technique for the isolation of mitochondria from lung tissue. We extensively described the technical difficulties concerning tissue quantity, tissue characteristics, tissue adjunct components, time of isolation, and the use of proteinases. Additionally, we described the experimental determination of several mitochondrial functional characteristics, providing information that might improve the reproducibility and analysis of lung tissue mitochondria. Ultimately, the method yielded a robust, maintained, and viable sample of pulmonary mitochondria.

## Data Availability Statement

The raw data supporting the conclusions of this article will be made available by the authors, without undue reservation.

## Ethics Statement

The animal study was reviewed and approved by the UFRJ–local Institutional Animal Care and Use Committee (015/17). Written informed consent was obtained from the owners for the participation of their animals in this study.

## Author Contributions

LM, WZ, and JN: conception and design, data acquisition, analysis and interpretation of data, drafting or revising the article, and contribution with reagents. DC, DO, and JC-d-A: data acquisition, and analysis and interpretation of data. LM was the principal investigator. All the authors discussed the results, commented on the manuscript, and approved the final version of the manuscript.

## Conflict of Interest

The authors declare that the research was conducted in the absence of any commercial or financial relationships that could be construed as a potential conflict of interest.

## Publisher’s Note

All claims expressed in this article are solely those of the authors and do not necessarily represent those of their affiliated organizations, or those of the publisher, the editors and the reviewers. Any product that may be evaluated in this article, or claim that may be made by its manufacturer, is not guaranteed or endorsed by the publisher.
